# Genetic Insights into the Comorbidity of Congenital Heart Disease and Neurodevelopmental Disorders: Focus on Copy Number Variations

**DOI:** 10.3390/jcdd13100529

**Published:** 2026-10-21

**Authors:** Ziyi Ye, Yingzhong He, Jing Guo, Jiajia Meng, Yufei Xu, Yingyan Wang, Bo Wang

**Affiliations:** 1 Pediatric Translational Medicine Institute, Shanghai Children’s Medical Center, Shanghai Jiao Tong University School of Medicine, Shanghai 200127, China; zoeyeziyi0322@163.com (Z.Y.); gjjing1020@163.com (J.G.); kcrown@yeah.net (J.M.); 2 Department of Neurology, Shanghai Children’s Medical Center, Shanghai Jiao Tong University School of Medicine, Shanghai 200127, China; heyingzhong@hotmail.com (Y.H.); xuyufei@scmc.com.cn (Y.X.); 3 NHC Key Laboratory of Birth Defects Prevention, Institute of Reproductive Health, Henan Academy of Innovations in Medical Science, Zhengzhou 451163, China

**Keywords:** congenital heart disease, neurodevelopmental disorders, copy number variations, gene dosage, non-coding regulation, three-dimensional genome, phenotypic heterogeneity

## Abstract

Congenital heart disease (CHD) and neurodevelopmental disorders (NDDs) frequently co-occur, indicating that cardiac and neural phenotypes can arise from shared developmental disturbances rather than exclusively from the physiological consequences of heart disease. Copy number variations (CNVs) provide a major genomic basis for this overlap, but their effects extend beyond changes in protein-coding gene dosage. This review synthesizes evidence from recurrent CNV syndromes, genomic cohorts, developmental studies, and experimental models to define how CNVs jointly influence heart and brain development. We examine four interconnected mechanisms: dosage alteration of pleiotropic and multiple genes, disruption of non-coding regulatory elements, remodeling of non-coding RNA networks, and reorganization of three-dimensional chromatin architecture. We further describe how developmental timing, cell-type-specific regulatory context, additional genomic variants, cardiac physiology, and clinical exposures shape incomplete penetrance and variable expressivity. Together, these findings support a layered model in which a primary CNV establishes cross-organ susceptibility, whereas genetic and developmental modifiers determine whether CHD, NDD, or both become clinically manifest. Clinically, CNV diagnosis can inform neurodevelopmental surveillance, multisystem management, and genetic counseling. Genotype-first longitudinal cohorts, developmental multi-omics, parallel cardiac and neural models, and prospective clinical studies will advance individualized risk prediction and intervention.

## 1. Introduction

Congenital heart disease (CHD) refers to a structural abnormality of the heart and/or great vessels that is present at birth [1]. It is among the most common birth defects, with a reported global birth prevalence of 9.41 per 1000 live births in 2010–2017 [2]. Advances in prenatal diagnosis, neonatal intensive care, catheter-based intervention, and cardiac surgery have substantially improved survival. In a nationwide Swedish cohort, survival among children with CHD born between 2010 and 2017 reached 97%, and most were projected to survive to adulthood [3]. Consequently, the clinical burden of CHD has shifted from predominantly early mortality toward lifelong morbidity and functional outcomes. Neurodevelopmental and psychosocial health have therefore become increasingly important determinants of long-term well-being and quality of life in the growing population of children and adults living with CHD [4].

Neurodevelopmental disorders (NDDs) comprise a heterogeneous group of disorders arising during development, including intellectual disability (ID), autism spectrum disorder, attention-deficit/hyperactivity disorder (ADHD), communication disorders, specific learning disorder, and motor disorders such as developmental coordination disorder and tic disorders [5]. Population-based studies and meta-analyses consistently demonstrate poorer cognitive and executive functioning in children with CHD than in the general population, although outcomes vary substantially across cardiac subtypes and study populations [6,7]. CHD is also associated with an increased risk of clinically diagnosed NDDs. A nationwide longitudinal study reported elevated risks of both ADHD and autism spectrum disorder among children with CHD [8]. Importantly, the neurodevelopmental phenotype is dynamic and age-dependent. Higher-order difficulties in executive, academic, behavioral, and psychosocial functioning may become more apparent as developmental demands increase, and neurocognitive difficulties can persist into adulthood [4]. These consequences extend beyond neuropsychological test performance: population-based and registry studies have identified differences in educational attainment, employment, and economic self-sufficiency among adults with CHD, particularly in those with more complex disease [9]. Together, these observations support a life-course view of neurodevelopmental morbidity in CHD rather than one restricted to early childhood.

The neurodevelopmental outcomes of individuals with CHD are shaped by multiple interacting factors. Altered fetal cerebral oxygen and nutrient delivery, placental and circulatory abnormalities, prematurity, perioperative neurological injury, prolonged hospitalization, and socioeconomic and family-related factors can all contribute to adverse developmental trajectories [4]. Nevertheless, measured patient-specific, preoperative, and perioperative factors explain less than one-third of the substantial interindividual variability in early neurodevelopmental outcomes after infant cardiac surgery, leaving much of the observed variation unexplained [10]. These observations have shifted attention toward intrinsic biological susceptibility, particularly genetic factors that may influence both cardiac and neural development before surgical or other postnatal exposures occur. Human genetic studies provide direct support for such shared susceptibility. Damaging de novo variants are enriched among individuals with CHD accompanied by neurodevelopmental or other extracardiac abnormalities, and many implicated genes are involved in chromatin regulation and developmental processes relevant to both cardiac and neural development [11,12]. More recent analyses further suggest that potentially damaging variants in specific functional gene categories, including chromatin-modifying and constrained genes, may contribute to variation in neurocognitive and behavioral outcomes among individuals with CHD [13]. Together, these findings support overlapping genetic and developmental mechanisms underlying at least a subset of cardiac and neurodevelopmental phenotypes.

Among the diverse genetic contributors to CHD, copy-number variants (CNVs) are particularly informative for understanding cardiac–neurodevelopmental co-occurrence [14,15]. CNVs can affect cardiac and neural development through three major mechanisms: altering the dosage of protein-coding genes, disrupting non-coding regulatory elements and gene expression, and reorganizing three-dimensional chromatin architecture and enhancer–promoter interactions [16]. These effects may involve multiple cardiac- and neurodevelopmentally relevant genes and regulatory elements within the same genomic event. Consistent with these properties, CNV syndromes frequently combine cardiac abnormalities with neurodevelopmental or neurocognitive phenotypes, and a systematic review identified substantial overlap between CNVs associated with congenital heart defects and those associated with neurocognitive impairment [14,15]. Clinical chromosomal microarray studies provide complementary evidence: CNVs associated with neurodevelopmental disorders can already be identified in neonates with CHD [17], and pathogenic CNVs linked to neurodevelopmental impairment have been documented across clinically ascertained CHD cohorts [18]. Thus, CNVs provide a genomic framework that integrates gene-dosage effects, multigene perturbation, regulatory disruption, and three-dimensional genome reorganization in the pathogenesis of CHD-NDD co-occurrence. This review examines the architecture and human genetic evidence of CNVs in CHD-NDD co-occurrence, the molecular and developmental mechanisms linking CNVs to cardiac and neural phenotypes, the determinants of incomplete penetrance and phenotypic heterogeneity, and the implications of CNV findings for neurodevelopmental surveillance, clinical management, and future mechanistic research.

## 2. CNV Architecture and Genetic Evidence in CHD-NDD Co-Occurrence

### 2.1. CNV Architecture and Clinical Interpretation

CNVs are structural variants that alter the copy number of genomic segments, most commonly through deletions or duplications, and collectively account for a substantial proportion of human genomic variation [19,20]. Their consequences depend not simply on the amount of DNA gained or lost, but on the genomic architecture of the affected interval, its gene and regulatory content, breakpoint location, and inheritance. Based on breakpoint architecture and formation mechanism, CNVs are broadly classified as recurrent or non-recurrent.

Recurrent CNVs typically arise at genomic loci flanked by highly homologous low-copy repeats (LCRs) or segmental duplications (SDs), which predispose to non-allelic homologous recombination (NAHR) during meiosis and generate rearrangements with similar breakpoints across unrelated individuals [19,21]. In contrast, non-recurrent CNVs show variable sizes and patient-specific breakpoints and are generated predominantly through non-homologous end joining or replication-based mechanisms, including fork stalling and template switching and microhomology-mediated break-induced replication [21]. Therefore, recurrent CNVs tend to produce recognizable genomic disorders with relatively stereotyped intervals, whereas non-recurrent CNVs generate more heterogeneous combinations of coding genes and regulatory elements, making genotype–phenotype interpretation substantially more challenging.

Importantly, genomic architecture, inheritance, and clinical pathogenicity represent distinct dimensions of CNV classification. Both recurrent and non-recurrent CNVs may arise de novo, be inherited from a parent, or occur in mosaic form. Under the ACMG/ClinGen framework, constitutional CNVs are classified as pathogenic, likely pathogenic, variants of uncertain significance (VUS), likely benign, or benign on the basis of multiple lines of evidence, including established dosage-sensitive genes or regions, overlap with previously characterized CNVs, population frequency, case and segregation data, inheritance, and phenotypic concordance [22]. No single feature is sufficient to determine pathogenicity: CNV size is informative, but not decisive; de novo occurrence strengthens but does not independently establish pathogenicity; and inheritance from an apparently unaffected parent does not necessarily indicate benignity, particularly for CNVs with incomplete penetrance or variable expressivity.

This multidimensional interpretation is particularly relevant to CHD-NDD co-occurrence, because a single CNV may encompass multiple dosage-sensitive genes and regulatory elements that contribute differently to cardiac and neurodevelopmental phenotypes. Accordingly, clinical interpretation should distinguish the pathogenicity of the CNV itself from its ability to predict a specific cardiac or neurodevelopmental outcome. This distinction is essential when evaluating CNVs with variable expressivity, age-dependent neurodevelopmental manifestations, or inheritance from apparently unaffected parents.

### 2.2. CNV Burden and Genetic Evidence for CHD-NDD Co-Occurrence

Rare CNVs are enriched in individuals with CHD, indicating that structural variation constitutes an important component of the genetic architecture of CHD. This enrichment is not restricted to clinically recognizable genomic syndromes. In a prospective study of 422 children clinically classified as having non-syndromic CHD, potentially pathogenic CNVs were identified in 12.1% of patients, compared with 5.0% of healthy pediatric controls [23]. CNV-defined cohorts provide more direct population-level evidence for CHD-NDD co-occurrence. A 20-year population-based retrospective study in Wales registered 1113 individuals with CNV-associated congenital abnormalities, of whom 785 were included in the analysis, representing 28 rare CNVs previously associated with CHD [15]. Among CNV carriers, more than one-third of individuals with CHD also had a recorded NDD. Across different CNV types, the proportion of individuals with CHD was significantly correlated with the proportion showing combined CHD-NDD phenotypes (95% CI (0.4062, 0.8449), *p* < 0.0001, *r* = 0.6829) [15]. However, this CNV-type-level association should be interpreted cautiously because of incomplete NDD ascertainment and small sample sizes for some CNV types, and does not establish causality or a shared mechanism. It does not establish causality or a shared developmental mechanism and should not be used to predict individual-level risk. Cardiac and neurodevelopmental phenotypes showed particularly prominent co-occurrence in genomic disorders such as 22q11.2 and 7q11.23 deletions [24,25], while global developmental delay (DD) was among the commonly recorded neurodevelopmental outcomes.

Neonatal chromosomal microarray analysis (CMA) studies further indicate that genetic risk can be identified before many neurodevelopmental phenotypes become clinically apparent. In a single-center study of 367 neonates with CHD who underwent CMA, 135 had abnormal findings, of whom 41 (30.4%) carried genomic abnormalities associated with NDDs that were detectable only by CMA [17]. In another study involving 525 neonates with CHD, 287 underwent CMA; pathogenic CNVs were identified in 61 (21.3%), and 48 of these 61 CNVs (78.7%) were associated with neurodevelopmental impairment [18]. These findings suggest that a subset of children with CHD already harbor genetically identifiable susceptibility to adverse neurodevelopmental outcomes at birth. However, such genetic susceptibility should not be equated with an observed neurodevelopmental outcome; rather, it may help identify infants who could benefit from early developmental surveillance and timely intervention to potentially mitigate adverse outcomes.

Systematic analyses across CNV syndromes provide further support for this pattern. A systematic review and meta-analysis including 16 studies across 12 CNV syndromes found recurrent co-occurrence of CHD and neurocognitive or neurodevelopmental abnormalities across multiple CNV disorders [14]. CNV characteristics, including size and inheritance, may be associated with the combined phenotypic burden, while certain CHD subtypes were also associated with more severe neurocognitive impairment [14]. However, substantial heterogeneity in ascertainment, definitions of neurodevelopmental outcomes, age at assessment, and study design limits direct comparisons of co-occurrence rates or NDD penetrance across different CNV loci.

### 2.3. Representative CNVs Associated with Cardiac-Neurodevelopmental Phenotypes

Several recurrent and well-characterized CNV syndromes provide particularly informative human models of CHD-NDD co-occurrence. Table 1 summarizes the cardiac and neurodevelopmental phenotypes reported in published cohorts of individuals carrying these CNVs. These loci differ substantially in population frequency, penetrance, and clinical impact; their presentation in a common table format does not imply equivalent clinical risk. The cohorts summarized in the table differ in study design, ascertainment, assessment age, and outcome definitions, limiting cross-locus comparisons. Across these syndromes, both cardiac and neurodevelopmental manifestations have been documented, with CHD and NDD co-occurring in a subset of affected individuals [14]. However, the involvement of both the heart and brain at the syndrome level does not imply that both phenotypes will occur in every carrier, as their expression is variable and depends on the CNV locus.

Comparison across these loci further shows that CHD-NDD co-occurrence does not follow a single genetic pattern. The 22q11.2 deletion exemplifies broad developmental susceptibility in which cardiac and neurodevelopmental phenotypes frequently coexist but show marked variable expressivity [25]. Reciprocal 1q21.1 rearrangements demonstrate that both CHD and NDD can occur in deletion and duplication carriers, although the two dosage states produce overlapping rather than simple mirror phenotypes [26,27]. Both CHD and DD or ID have been reported in carriers of 8p23.1 deletions and duplications [28,29]. In contrast, the 7q11.23 deletion provides a clear contiguous-gene model in which cardiovascular and neurodevelopmental manifestations coexist because different dosage-sensitive genes within the same CNV preferentially contribute to the two phenotypic domains [30]. Together, these locus-specific patterns indicate that CHD-NDD co-occurrence can arise through different configurations of dosage-sensitive genomic content rather than a single uniform genetic architecture. These human genetic observations provide the basis for examining the molecular and developmental mechanisms that connect cardiac and neurodevelopmental phenotypes in the following section.

**Table 1:** Cardiac and neurodevelopmental phenotypes associated with selected CNV syndromes.

CNV Syndrome	CHD Phenotypes	CHD (%)	Neurodevelopmental and Psychiatric Phenotypes	NDD (%)	Co-Occurrence	*n*	Ref
1p36 deletion	VSD; PDA; atrial septal defect (ASD); TOF; overriding aorta; abdominal aortic hypoplasia	36/77, 46.7%	ID; DD, language delay, motor delay, developmental regression; behavioral disorders	75/75, 100%	NR	86	[31]
1q21.1 deletion	PFO, PDA, Ascending aorta dilatation	6/20, 30%	ID, ADHD; speech delay, fine motor delay, gross motor delay, learning difficulties; bipolar disorder	12/20, 60%	3/20, 15%	20	[32]
1q21.1 duplication	TOF, PDA, ASD, PFO	6/16, 37.5%	ID, ADHD; speech delay, gross motor delay, fine motor delay, verbal apraxia; bipolar disorder	9/16, 56.2%	2/16, 12.5%	16	[32]
1q41q42 microdeletion	VSD	9/31	ID; DD	24/31	7/31	31	[33]
7q11.23 deletion (Williams-Beuren syndrome)	SVAS; main/branch pulmonary artery stenosis; occasional septal defects	198/231, 85.7%	Mild–moderate ID, ADHD; DD, visuospatial deficits; hypersociability	167/182, 91.8%	NR	231	[34]
7q11.23 duplication	PDA; ASD; VSD; subvalvar aortic stenosis	10/53, 18.9%	Autism (18.9%), ADHD (37.0%), Developmental Coordination Disorder (74.2%), Speech Sound Disorder (82.6%)	NR	NR	53	[35]
8p23.1 deletion	ASD; CAVC; pulmonary valve stenosis; LSVC; heterotaxy-spectrum defects; CoA; HLHS; VSD; Ebstein anomaly; AVS; abnormal pulmonary venous return	16/18, 88.9%	ID, autism spectrum disorder, ADHD; DD	55.6%, 10/18	50%, 9/18	18	[36]
9q34.3 Subtelomeric deletion (Kleefstra syndrome)	ASD, VSD, PS, BAV, PFO	5/16, 31%	ID; DD, speech delay/absent speech, motor developmental delay	16/16, 100%	5/16, 31.25%	16	[37]
11q terminal deletion (Jacobsen syndrome)	HLHS; Shone’s complex; CoA; VSD; Valve abnormalities	21/27, 77.8%	ID, autism spectrum disorder, ADHD; DD, expressive language impairment, gross/fine motor delay	19/23, 82.6%	NR	29	[38]
15q24 microdeletion	PDA; PPS; PS	2/7 ^a^, 28.6%	ID, autism spectrum disorder; DD, expressive speech delay/absent speech	15/15, 100%	2/7 ^a^, 28.6%	15	[39]
15q11.2 BP1-BP2 deletion	VSD, TOF, CoA, ASD, dextrocardia, PA hypoplasia, APVD, valvular PS	9/52, 17.3%	ID (63%), autism spectrum disorder (9.8%), ADHD (48.8%); speech impairment (85.4%), motor delay (43.9%), learning difficulties (65.9%); OCD (4.9%)	Overall percentage NR	5 cases mentioned	52 ^b^	[40]
16p11.2p12.2 microdeletion	Ebstein anomaly; PS	1 case mentioned	ID; DD	4/4, 100%	1 case mentioned	4	[40]
22q11.2 deletion	TOF, VSD, IAA, PA/VSD, TrA, PVS, ASD, AVSD, TGA, DAA, APV; AVS; aortic valve hypoplasia, CoA, DORV, atrial isomerism, HLHS; PDA, tricuspid valve hypoplasia	409/545 ^c^, 75%	ID, ADHD; DD, speech delay, mild/moderate/severe motor developmental delay, learning difficulties	268/33, 879.3%	NR	558	[41]
22q11.2 duplication	Arteria lusoria, AVSD, TGA, CoA/hypoplastic aortic arch, secundum ASD, PDA	3/12, 25%	ID, ADHD, autism spectrum disorder, DCD; DD, speech-language delay, motor delay, learning problems	11/11, 100%	11 cases mentioned	13	[42]
22q13 microdeletion (Phelan-McDermid syndrome)	PDA, VSD, BAV, PLSVC, TAPVR, CoA	30/189, 15.9%	ID (74.3%), autism spectrum disorder (62.9%); DD (95.8%), speech delay (97.4%)	NR	NR	187	[43]

^a^: Seven of the 15 individuals underwent cardiac assessment. ^b^: Twenty-six of the 52 individuals underwent cardiac assessment. ^c^: Cardiac status was documented for 545 of the 558 individuals. In the “Neurodevelopmental and psychiatric phenotypes” column, semicolons separate NDD diagnoses, developmental/cognitive findings, and behavioral/psychiatric manifestations. Co-occurrence denotes individuals with both CHD and NDD among all carriers, or among those assessed for both phenotypes when data are incomplete; denominators are specified by row. Frequencies derive from heterogeneous, often small clinical cohorts, do not necessarily represent diagnostic rates or population-based penetrance, and should not be directly compared across CNV loci. NR, not reported or not determinable from the source.

## 3. Molecular and Developmental Mechanisms Linking CNVs to Cardiac-Neurodevelopmental Phenotypes

### 3.1. Protein-Coding Gene Dosage: Haploinsufficiency, Triplosensitivity, and Multigene Effects

Protein-coding gene dosage imbalance is a central mechanism linking CNVs to combined cardiac and neurodevelopmental phenotypes. Copy-number loss or gain can disrupt dosage-sensitive developmental programs through two non-exclusive routes: altered dosage of a pleiotropic gene may affect both cardiac and neural development, whereas a multigene CNV may simultaneously alter genes with distinct organ-specific functions.

The 22q11.2 deletion illustrates the multigene mechanism. Reduced dosage of *TBX1* and *CRKL* disrupts second heart field and cardiac neural crest-dependent programs, contributing to conotruncal and aortic arch abnormalities [25,44]. Within the same interval, *DGCR8* haploinsufficiency alters microRNA processing and dysregulates pathways involved in neuronal excitability, calcium signaling, and synaptic function [45]. A similar organization occurs in the 7q11.23 deletion underlying Williams–Beuren syndrome: *ELN* haploinsufficiency drives supravalvular aortic stenosis and related vascular abnormalities, whereas reduced dosage of *GTF2I*, *GTF2IRD1*, and other interval genes contributes to the characteristic cognitive and behavioral phenotype [24]. At 1q21.1, altered *GJA5* dosage has been implicated in cardiac abnormalities, while dosage changes involving *NOTCH2NL* may affect cortical neurogenesis [26,27]. These loci show how a single CNV can perturb complementary cardiac and neurodevelopmental genes.

Reciprocal duplications demonstrate that dosage gain can likewise affect both developmental systems. In 7q11.23 duplication syndrome, increased *GTF2I* dosage alters cortical progenitor proliferation and neuronal differentiation, producing autism-related behavioral phenotypes [46]. Above that, increased *ELN* dosage may contribute to ascending aortic dilatation, while structural cardiac defects occur in a subset of carriers [24]. At 22q11.2, evidence from animal models indicates that both loss of *Tbx1* expression (haploinsufficiency) and overexpression (triplosensitivity) result in comparable outflow tract developmental anomalies, suggesting a strict dosage-dependent mechanism [47]. Carriers may also exhibit cognitive, speech, and other neurodevelopmental abnormalities, which can coexist with CHD [48].

**Figure 1:**
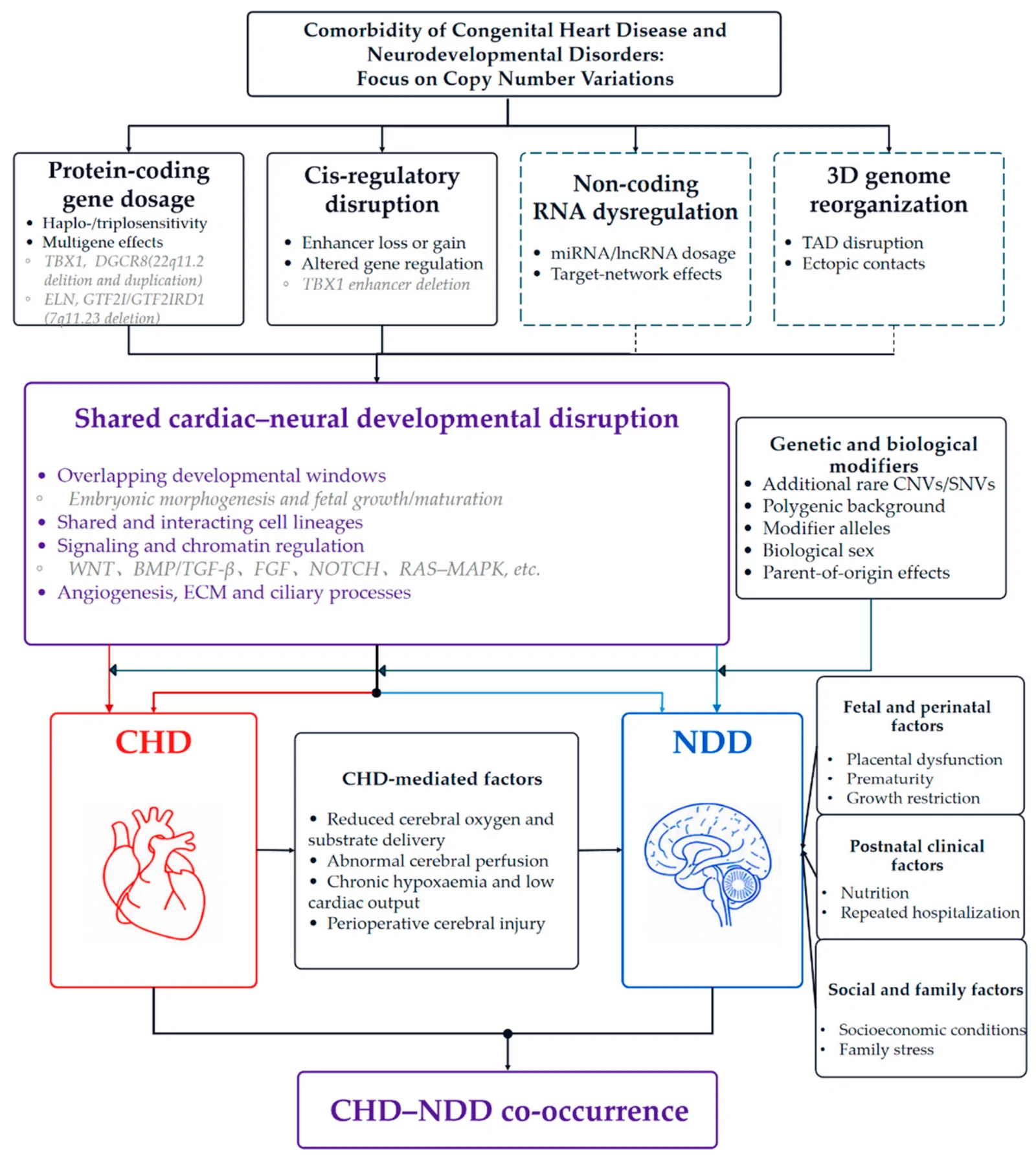
Proposed pathways linking CNVs to CHD-NDD co-occurrence. CNVs may affect cardiac and neural development through altered protein-coding gene dosage, cis-regulatory disruption, non-coding RNA dysregulation, and three-dimensional genome reorganization. Genetic and biological modifiers influence phenotypic expression, while CHD-related alterations in cerebral physiology and additional developmental, clinical, and social factors may contribute to neurodevelopmental outcomes. Solid-bordered boxes indicate mechanisms or contributing factors supported by evidence discussed in the main text; dashed-bordered boxes indicate hypothesized mechanisms for which direct evidence remains limited in the context of CHD-NDD co-occurrence. Filled bullets (•) summarize mechanisms or contributing factors, whereas open circles (○) and gray text identify illustrative examples discussed in the main text. These examples do not imply that each independently causes both CHD and NDD, and the strength of evidence varies across mechanisms and loci.

CNV-associated CHD-NDD co-occurrence therefore primarily reflects the combined effects of multiple dosage-sensitive genes within the same interval (Figure 1). However, the cross-organ effects of individual genes remain insufficiently characterized and require validation in integrated cardiac and neural models.

### 3.2. Regulatory and Three-Dimensional Genome Disruption

Beyond changing protein-coding gene dosage, CNVs can alter developmental transcription through disruption of cis-regulatory elements, changes in non-coding RNA dosage, and reorganization of chromatin architecture. These mechanisms extend the functional effects of a CNV beyond the genes directly deleted or duplicated and may generate cardiac and neurodevelopmental phenotypes through shared or lineage-specific regulatory programs.

Cis-regulatory disruption. Enhancers and insulators control the timing, location, and magnitude of developmental gene expression; their deletion, duplication, or displacement can therefore alter a target gene while leaving its coding sequence intact [49,50,51]. At the 22q11.2 locus, Gao et al. performed whole-genome sequencing in 428 patients with tetralogy of Fallot (TOF), identifying 31 patients with 22q11.2 deletions encompassing *TBX1* as well as seven functional non-coding variants in a *TBX1* enhancer. Deletion of this enhancer in embryonic stem cells reduced *TBX1* expression in blood vessel organoids, impaired angiogenesis and pericyte coverage, altered tight-junction morphology, and disrupted angiogenesis-related transcriptional programs involving *DLL4* and *TGFBR2*. *TBX1* re-expression restored the expression of these target genes and improved tight-junction formation and pericyte coverage [52]. These findings provide functional evidence that disruption of a regulatory element at a major CNV-syndrome locus can contribute to the associated cardiac phenotype. More broadly, they raise the possibility that a CNV encompassing enhancers active in cardiac and neural lineages could contribute to CHD and NDD through parallel, tissue-specific regulatory effects.

Non-coding RNA dosage. Lu et al. analyzed 568 lncRNAs located within recurrent non-syndromic CHD-associated CNVs using transcriptomic data from 313 human developmental samples and subsequently extended the analysis to 1500 lncRNAs within non-syndromic and syndromic CHD-associated CNVs. Distinct CNV-lncRNA modules were associated with the developing heart, brain, and cerebellum. One module was enriched for both CHD genes and autism-spectrum-disorder genes, while comparison of 50 heart and 87 brain samples identified tissue-biased CNV-lncRNA expression [53]. In an independent analysis of CNVs from 743 patients with CHD, Penaloza et al. identified 18 candidate lncRNAs in heart-associated regulatory modules, including transcripts co-expressed with *NKX2-5* [54]. These studies connect CNV-associated lncRNAs to cardiac development and place subsets of them within regulatory networks relevant to both CHD and NDD.

Three-dimensional genome reorganization. Zhang et al. generated Hi-C maps from lymphoblastoid cell lines derived from five 22q11.2 deletion carriers and six controls and integrated these data with RNA sequencing, histone-mark profiling, and CTCF binding [55]. The deletion reduced contacts involving the deleted interval, increased interactions between its flanking regions, altered topological domains, and produced broader changes in chromosome folding and gene expression. Analysis of two 1q21.1 deletion cell lines revealed a comparable pattern of local and genome-wide contact changes [55]. The transcriptional consequences of such spatial relationships are also illustrated by the 16p11.2 locus. Blumenthal et al. performed RNA sequencing of cerebral cortex from four deletion, four duplication, and eight wild-type mice, together with lymphoblastoid cell lines from 34 members of seven multiplex autism families carrying 16p11.2 rearrangements [56]. In addition to dosage-dependent expression within the CNV, they identified cis- and trans-effects at distal loci. Integration with independent Hi-C data showed that trans-regulated regions were enriched among loci that physically interact with 16p11.2 in the nucleus. The affected networks included synaptic genes such as *NRXN1* and *NRXN3*, chromatin regulators including *CHD8*, *EHMT1*, and *MECP2*, and neurodevelopmental genes such as *TCF4*, *SATB2*, and *FMR1* [56].

Together, these studies establish cis-regulatory disruption, altered non-coding RNA dosage, and three-dimensional genome reorganization as substantive components of CNV pathogenicity. Cis-regulatory studies provide direct evidence for cardiac effects, CNV-lncRNA analyses connect cardiac and neurodevelopmental networks, and chromatin-conformation studies define spatial reorganization with functional consequences for neurodevelopment. These mechanisms complement protein-coding gene dosage and extend the developmental influence of a CNV beyond the genes located within the rearranged interval. However, they have not yet been integrated into a causal model of CHD-NDD co-occurrence: no study has demonstrated that a specific non-coding or three-dimensional alteration induced by the same CNV disrupts cardiac and neural development in parallel.

### 3.3. Developmental and Spatiotemporal Convergence of Heart and Brain

The contribution of CNVs to CHD-NDD co-occurrence is rooted in the temporal overlap and molecular interdependence of heart and brain development. The first and second heart fields are established at approximately post-conception day 15, the primitive heart tube begins rhythmic contraction around day 21, and cardiac looping, outflow-tract formation, and septation produce a largely mature cardiac structure by approximately day 50 [57]. In parallel with cardiac morphogenesis, neural tube formation and regional brain patterning begin during early embryogenesis. Neural progenitor proliferation and neurogenesis then proceed predominantly during fetal development, and neuronal migration is concentrated in early and mid-gestation, whereas synaptogenesis, myelination, and activity-dependent refinement of neural circuits extend into early adulthood [58,59,60]. A CNV perturbing this shared developmental window can therefore produce an early structural cardiac defect while initiating neurodevelopmental alterations that become progressively apparent with brain maturation.

Cellular convergence reflects the reuse of common developmental operations rather than identity between mature cardiac and neural cell types. Although cranial and cardiac neural crest populations have distinct developmental contributions, they share substantial transcriptional and enhancer-regulatory features [61]. Cardiac neural crest cells migrate through the pharyngeal arches and contribute to outflow-tract septation and aortic-arch remodeling through interactions with second heart field progenitors and pharyngeal tissues [62]. Shared regulators can influence these populations and neural progenitors through distinct cellular effects. *CHD7* illustrates this principle: it regulates early neural crest formation and migration [63], supports cardiac neural crest differentiation [64], modulates second heart field gene expression [65], and maintains neuroepithelial lineage identity [66]. Endothelial lineages provide a complementary mechanism through tissue interactions. Endocardial signaling regulates myocardial growth and morphogenesis, whereas cerebral vascular endothelial cells form the developing brain vasculature and influence neural progenitor proliferation and differentiation through neurovascular interactions and oxygen delivery [67]. Together, these findings provide a plausible basis for CNVs to affect cardiac and neural development through shared regulation and lineage-specific interactions.

At the molecular level, heart and brain development repeatedly employ WNT, BMP/TGF-β, FGF, NOTCH, and RAS–MAPK signaling to regulate progenitor maintenance, lineage specification, migration, and tissue patterning [60,68]. These signaling systems converge with three additional developmental processes. First, vasculogenesis and angiogenesis coordinate cardiovascular morphogenesis with expansion of the cerebral vascular network, linking endothelial development to the growth and metabolic support of neural tissue [69]. Second, the extracellular matrix provides both mechanical and biochemical cues: it guides cardiac progenitor migration, cushion and valve formation, and chamber morphogenesis [70], while regulating neural progenitor behavior, neuronal migration, axon extension, and neural tissue architecture [71]. Third, motile and primary cilia translate morphogen and mechanical signals into developmental responses. Ciliary function is required for left–right patterning and cardiac morphogenesis and also regulates neural progenitor proliferation, neuronal migration, and brain patterning [72]. Direct functional evidence for this intersection was provided by Teerikorpi et al., who identified 45 CHD-associated genes that altered neural progenitor proliferation or survival; shared CHD–autism genes were enriched in ciliary biology, and disruption of *TAOK1* affected both brain and heart development in *Xenopus* [73]. CNVs that alter multiple components of these interconnected processes can therefore propagate a single dosage imbalance across cardiac and neural lineages. Spatiotemporal convergence thus explains both the co-occurrence of cardiac and neurodevelopmental phenotypes and the interval between an early structural cardiac diagnosis and the later emergence of neurodevelopmental impairment.

## 4. Determinants of Phenotypic Heterogeneity in CNV Carriers

### 4.1. Incomplete Penetrance and Variable Expressivity

Incomplete penetrance and variable expressivity are defining features of CNV-associated disorders. Carriers of the same CNV may present with CHD, NDD, both phenotypes, or neither, and the severity of each manifestation can differ markedly. This diversity reflects a probabilistic relationship between genomic dosage and developmental outcome. A pathogenic CNV establishes susceptibility across one or more organ systems, whereas its phenotypic expression depends on the functional content of the affected interval and the biological context in which the dosage change acts.

The distribution of cardiac and neurodevelopmental phenotypes differs across CNV loci. The 22q11.2 deletion produces a broad developmental spectrum in which CHD and NDD frequently coexist but vary in type and severity [25]. Reciprocal 1q21.1 rearrangements generate overlapping rather than mirror-image phenotypes, demonstrating that deletion and duplication do not produce simple opposing effects [26,27]. Similarly, the 7q11.23 deletion combines characteristic cardiovascular and neurodevelopmental manifestations, while their severity remains variable among carriers [24]. Large cohort analyses across genomic disorders further show that recurrent CNVs differ in inheritance, penetrance, and the frequency of additional genomic variants [74,75]. Cardiac and neurodevelopmental penetrance are thus related at the locus level but only partially concordant within individuals.

### 4.2. Genetic, Clinical, and Developmental Modifiers

The first layer of phenotypic modification resides in the CNV itself. Dosage direction, breakpoint location, mosaicism, and the coding and regulatory content of the affected interval determine which developmental processes are disrupted [19,20,21]. Beyond altering linear gene dosage, CNVs can perturb three-dimensional genome organization by disrupting topologically associating domain boundaries, changing enhancer–promoter contacts, or creating ectopic regulatory interactions, thereby modifying the timing, location, and magnitude of gene expression during cardiac and neural development [76,77]. The broader genetic background constitutes a second layer of modification. In 16p12.1 deletion carriers, additional large CNVs were associated with greater developmental severity and a broader spectrum of congenital abnormalities, supporting a two-hit model of variable phenotypic expression [78]. Similarly, among 2312 children ascertained for ID, DD, or congenital anomalies, 10.1% carried a second large CNV, and multiple genomic alterations were associated with greater overall phenotypic complexity [75]. Evidence from phenotype-specific studies also indicates that genetic background can modify each component of the CHD-NDD phenotype, although these effects have been examined separately [79,80,81,82]. Together, these findings position the primary CNV as a major determinant whose effects are amplified or buffered by the surrounding genome.

Biological sex and parent of origin provide additional sources of phenotypic variation. Affected females with NDDs carry a greater burden of deleterious autosomal CNVs than affected males (odds ratio [OR] = 1.46, *p* = 8 × 10^−10^), consistent with a sex-dependent liability threshold, while the magnitude and direction of sex bias vary across CNV loci [83]. Sex-related modification has also been observed for cardiac manifestations: in Williams–Beuren syndrome, male carriers of the 7q11.23 deletion exhibit more severe supravalvular aortic stenosis and overall cardiovascular disease than female carriers [84]. Parent of origin may modify effective gene dosage when CNVs involve imprinted loci or parent-biased allelic regulation. This principle is well established within the 15q11–q13 Prader–Willi/Angelman critical region, where loss of paternal expression of genes and transcripts including *SNURF-SNRPN* and the *SNORD116* cluster causes Prader–Willi syndrome, whereas loss of maternally expressed *UBE3A* in neurons causes Angelman syndrome [85]. At the adjacent 15q11.2 BP1–BP2 interval, an exploratory study of 71 carriers reported parent-of-origin-associated phenotypic differences, with maternal deletions associated with selected neurodevelopmental features and paternal deletions with CHD or muscular phenotypes [86]. However, the four principal protein-coding genes within BP1–BP2 are generally considered biallelically expressed, and the molecular basis of these preliminary associations remains unresolved. Together, biological sex and parent-of-origin effects may modify the relative penetrance of cardiac and neurodevelopmental manifestations and thereby contribute to variation in the clinical expression of CHD-NDD co-occurrence.

Genomic susceptibility is subsequently expressed within a dynamic developmental and clinical context. Cardiac and neural development share overlapping embryonic periods, but dosage-sensitive genes act in distinct cell types, regulatory environments, and stages of maturation [4,16]. CHD can further alter brain development through impaired fetal cerebral oxygen and substrate delivery. Fetal MRI studies have linked reduced cerebral oxygen consumption in CHD to smaller brain volume [87], while neonatal imaging has demonstrated delayed brain maturation before cardiac surgery [88]. Prematurity, perioperative neurological injury, prolonged hospitalization, and socioeconomic conditions subsequently shape the neurodevelopmental trajectory [4,10]. CNVs can therefore connect CHD and NDD through two complementary routes: direct disruption of shared cardiac and neural developmental programs, and a sequential pathway in which genetically mediated cardiac disease alters subsequent brain development.

CNVs can confer genetic susceptibility to both CHD and NDD, with variable penetrance of the cardiac and neurodevelopmental manifestations. The occurrence of neurodevelopmental impairment in carriers without structural CHD supports a genetic contribution to NDD that does not depend on structural cardiac disease. These intrinsic risks may coexist with additional effects of CHD-related physiological disturbances and clinical exposures. Comparisons of carriers of the same recurrent CNV with and without structural CHD provide one approach to distinguishing these contributions. One study found no significant differences in psychiatric disorder prevalence or neurocognitive performance between 22q11.2 deletion carriers with and without CHD [89]. However, MRI studies associated CHD with additional reductions in cortical and hippocampal volumes, with less consistent effects on cognitive outcomes [90,91]. These findings suggest that the apparent contribution of CHD may differ across structural brain and functional outcomes. Longitudinal assessments integrating fetal and preoperative brain imaging, oxygenation, cerebral perfusion, perioperative injury, and subsequent developmental outcomes could further clarify the contribution of CHD-mediated pathways. However, residual confounding in clinical cohorts limits causal inference and complicates the separation of direct genetic effects from the effects of CHD-related physiological disturbances and perioperative exposures. The phenotype of an individual CNV carrier ultimately reflects the combined effects of the primary genomic alteration, genetic background, biological sex, parent-of-origin effect, developmental timing, and disease-related exposures. This layered architecture explains why CHD and NDD cluster among CNV carriers without producing uniform outcomes. It also defines the clinical value of CNV findings: identification of a developmental risk spectrum that can guide longitudinal surveillance and early intervention.

## 5. Clinical Translation of CNV Findings in CHD

A CNV diagnosis can do more than explain cardiac anatomy. By identifying a genomic disorder that spans cardiac, neurodevelopmental, and other organ systems, it can inform family testing, define developmental risk, and initiate surveillance before functional difficulties become clinically apparent. Its clinical value therefore depends on a continuous process: appropriate test selection, evidence-based interpretation, genotype-informed management, and counseling that clearly separates molecular diagnosis from individual prognosis.

### 5.1. Genetic Testing and CNV Interpretation

Genetic testing should be incorporated early when CHD occurs with dysmorphic features, impaired growth, extracardiac malformations, developmental abnormalities, or a family history suggestive of inherited disease. CMA remains a principal first-line assay for syndromic CHD because it detects genome-wide deletions and duplications that are not resolved by conventional karyotyping [22,92]. Prenatally, CMA is recommended when a major fetal structural abnormality is identified and invasive diagnostic testing is performed [93]. After a nondiagnostic CMA, trio-based exome or genome sequencing can identify sequence-level causes and, when supported by validated analytical pipelines, additional CNVs and structural variants, particularly in children with multiple congenital anomalies, DD, or ID [94]. The diagnostic pathway should thus be matched to the suspected variant class and the complexity of the phenotype. Constitutional CNVs should be evaluated using the ACMG/ClinGen five-tier system: pathogenic, likely pathogenic, uncertain significance, likely benign, or benign [22]. Classification integrates dosage direction, genomic coordinates, interval size, dosage-sensitive genes, regulatory content, population frequency, case-level enrichment, segregation, and inheritance.

### 5.2. Genotype-Informed Neurodevelopmental Surveillance and Intervention

A pathogenic CNV can move neurodevelopmental care from reactive referral to prospective risk management. The 2024 American Heart Association scientific statement identifies a genetic abnormality or syndrome associated with developmental disability as an independent neurodevelopmental risk factor in individuals with CHD. Developmental surveillance should begin when genomic risk is recognized and continue across the lifespan. For children with CHD at high neurodevelopmental risk, structured evaluations at approximately 6 months, 18 months, 3 years, and 5 years enable early assessment of motor function, language, cognition, adaptive behavior, and social development. Later evaluations should coincide with increasing cognitive and environmental demands, including entry into primary school, transition to middle school, adolescence, and preparation for adult care [4]. Maternal hyperoxygenation is being investigated as an experimental prenatal neuroprotective intervention for fetal CHD. Acute maternal hyperoxygenation can transiently increase MRI-derived cerebral oxygenation and alter fetal hemodynamics in selected CHD physiologies. However, these short-term physiological responses have not been shown to translate into improved brain growth, reduced structural brain injury, or better neurodevelopmental outcomes [95,96,97]. Evidence for the long-term safety and neurodevelopmental efficacy of repeated or prolonged administration remains limited.

CNV-specific phenotypes can refine both the timing and content of surveillance. Genomic disorders dominated by speech and language impairment justify early communication assessment; those associated with hypotonia, coordination difficulties, or feeding dysfunction support prompt motor and feeding evaluation. CNVs linked to autism, attention-deficit/hyperactivity disorder, anxiety, or psychosis require behavioral and psychiatric monitoring as these phenotypes become developmentally observable. The management of 22q11.2 deletion syndrome illustrates this approach: surveillance integrates developmental and cognitive assessment, evaluation for NDDs, and psychiatric monitoring with cardiac, immune, endocrine, auditory, palatal, and feeding assessment [98]. Such integration is particularly important in CHD-NDD comorbidity because hearing loss, sleep disturbance, seizures, endocrine dysfunction, and nutritional problems can intensify an underlying developmental vulnerability.

## 6. Future Directions: From Genetic Association to Mechanistic and Clinical Validation

CNV research in CHD must now move from locus-level association toward developmental prediction, experimental causality, and measurable clinical benefit. Three connected priorities define this transition: longitudinal characterization of CNV carriers, integration of clinical and developmental multi-omic data, and experimental testing of their causal contribution to CHD-NDD co-occurrence.

Longitudinal cohorts should follow CNV carriers identified prenatally, through clinical testing, and in population biobanks. Future cohorts should combine precise CNV structure, dosage direction, mosaicism, inheritance, additional rare variants, and genetic ancestry with standardized cardiac phenotyping and repeated, age-appropriate assessment of cognition, language, motor function, behavior, adaptive function, and psychiatric health. Cardiac physiology, placental findings, perioperative exposures, and socioeconomic conditions should be recorded within the same framework. Such cohorts would define age-dependent cardiac and neurodevelopmental penetrance, identify modifiers of CHD-NDD co-occurrence, and support models that predict developmental trajectories.

Integrated CNV–phenotype resources are equally important. Databases should connect exact genomic coordinates and breakpoint structures with longitudinal cardiac and neurodevelopmental phenotypes across diverse populations, enabling systematic reanalysis of CNVs of uncertain significance and the development of interpretable, AI-assisted classification models. Evidence-based CNV interpretation already depends on standardized dosage, population, case, and segregation data, while periodic incorporation of ClinGen dosage-sensitivity evidence can produce clinically meaningful reclassification [22,99]. Developmental single-cell and spatial atlases now resolve cell types and gene-expression programs in the embryonic human heart, whereas single-cell epigenomic and multi-omic studies define dynamic regulatory states across human cortical development [100,101]. Integrating these resources with CNV structure, enhancer–promoter connectivity, three-dimensional chromatin organization, proteomic effects, and patient phenotype would locate CNV consequences within the relevant cell type and developmental window. Computational prioritization should also incorporate haploinsufficiency, triplosensitivity, gene-level constraint, and additional rare-variant burden; large-scale cross-disorder analysis has already produced genome-wide probabilities of deletion and duplication intolerance [102].

Functional studies should then determine whether prioritized alterations exert causal effects across both organs. CRISPR-based approaches can reconstruct recurrent deletions and duplications in human iPSCs while preserving their multigene architecture [103], and an isogenic model has already been generated for the 22q11.2 A–B deletion [104]. Specific chromatin contacts can be experimentally manipulated [77], and studies of TAD disruption have linked altered enhancer–promoter interactions to developmental limb malformation [105]. These findings provide a rationale for extending such approaches to paired cardiac and neural iPSC/organoid models to test whether CNV-induced changes in three-dimensional chromatin organization contribute to CHD, NDD, and their co-occurrence. Patient-derived or engineered iPSCs should be differentiated in parallel into cardiac progenitors, cardiac neural crest cells, heart organoids, neural progenitors, neurons, and cortical organoids. Comparing CNV correction, single-gene dosage restoration, and targeted regulatory perturbation, with assessment of chromatin organization and developmental phenotypes in both lineages, would help distinguish dosage effects from shared non-coding or three-dimensional regulatory mechanisms. Existing models support the feasibility of this approach: 22q11.2 deletion cortical organoids exhibit altered neuronal activity and calcium signaling that can be reproduced and rescued through manipulation of *DGCR8* dosage [45], while self-assembling human heart organoids recapitulate heart-field specification, multicellular organization, chamber formation, vascular development, and functional activity [106].

Complementary in vivo studies should distinguish intrinsic neural effects of CNVs from secondary effects mediated by cardiac or placental dysfunction. Animal models that faithfully reproduce CNV structure, combined with tissue-specific genetic manipulation and controlled fetal-hypoxia paradigms, would help separate these contributions and assess the role of reduced fetal cerebral oxygen delivery [107,108,109]. Together with longitudinal clinical data and paired iPSC/organoid experiments, these studies would clarify how genetic and physiological mechanisms contribute to CHD-NDD co-occurrence.

## 7. Conclusions

CNVs provide a coherent genomic framework for understanding the frequent co-occurrence of CHD and NDD. Their pathogenic effects extend beyond the dosage of individual protein-coding genes to encompass multigene interactions, non-coding regulatory disruption, non-coding RNA dysregulation, and three-dimensional genome reorganization. These alterations converge on dosage-sensitive developmental programs that operate across cardiac and neural lineages. The resulting phenotype is further shaped by CNV structure, genetic background, developmental timing, cardiac physiology, and clinical exposures, accounting for the incomplete penetrance and variable expressivity observed among carriers.

Accordingly, a pathogenic CNV should be viewed not only as an explanation for cardiac anatomy but also as an indicator of broader developmental risk. Integrating CNV findings with longitudinal neurodevelopmental surveillance, multisystem assessment, and individualized genetic counseling can shift clinical care from retrospective diagnosis toward prospective risk management. The next advance will depend on linking genotype-first cohorts with developmental multi-omics and parallel cardiac–neural models, followed by prospective evaluation of clinical utility. This integrated framework can transform CNVs from descriptive genomic findings into actionable predictors of developmental trajectories and provide a mechanistic foundation for earlier, more precise intervention in individuals at risk of CHD-NDD comorbidity.

## Data Availability

No new data were created or analyzed in this study.

## Abbreviations

The following abbreviations are used in this manuscript:

ACMG	American College of Medical Genetics and Genomics
ADHD	Attention-deficit/hyperactivity disorder
AI	Artificial intelligence
APV	Absent pulmonary valve
AS	Aortic stenosis
ASD	atrial septal defect
AVSD	Atrioventricular septal defect
BAV	Bicuspid aortic valve
BMP	Bone morphogenetic protein
CAVC	Complete atrioventricular canal
CHD	Congenital heart disease
CMA	Chromosomal microarray analysis
CNV	Copy-number variant
CoA	Coarctation of the aorta
CRISPR	Clustered regularly interspaced short palindromic repeats
CTCF	CCCTC-binding factor
DAA	Double aortic arch
DCD	Developmental coordination disorder
DD	Developmental delay
DORV	Double-outlet right ventricle
d-TGA	Dextro-transposition of the great arteries
FGF	Fibroblast growth factor
Hi-C	High-throughput chromosome conformation capture
HLHS	Hypoplastic left heart syndrome
IAA	Interrupted aortic arch
ID	Intellectual disability
iPSC	induced pluripotent stem cell
LCR	Low-copy repeat
lncRNA	Long non-coding RNA
LSVC	Left superior vena cava
MAPK	Mitogen-activated protein kinase
MRI	Magnetic resonance imaging
MS	Mitral stenosis
NAHR	Non-allelic homologous recombination
NDD	Neurodevelopmental disorder
NR	Not reported
OCD	Obsessive–compulsive disorder
PA	Pulmonary atresia
PDA	Patent ductus arteriosus
PFO	Patent foramen ovale
PLSVC	Persistent left superior vena cava
PPS	Peripheral pulmonary stenosis
PS	Pulmonary stenosis
SD	Segmental duplication
SVAS	Supravalvular aortic stenosis
TAD	Topologically associating domain
TAPVR	Total anomalous pulmonary venous return
TGF-β	Transforming growth factor beta
TGA	Transposition of the great arteries
TOF	Tetralogy of Fallot
TrA	Truncus arteriosus
VSD	Ventricular septal defect
VUS	Variant of uncertain significance
